# Supplementary material for: Assessing CYP2C8-Mediated Pharmaceutical Excipient-Drug Interaction Potential: A Case Study of Tween 80 and Cremophor EL−35
Source: Pharmaceutics. 2021 Sep 17;13(9):1492. doi: 10.3390/pharmaceutics13091492 (PMC8467590; doi:10.3390/pharmaceutics13091492)
Supplement: Supplementary file 1 [file pharmaceutics-13-01492-s001.zip › pharmaceutics-1369924-SI.pdf]

pharmaceutics

Supplementary materials: Assessing CYP2C8-Mediated Pharmaceutical Excipient-Drug Interaction Potential: A Case Study of Tween 80 and Cremophor EL-35

Chengming Wen, Haoyang Hu, Wenwen Zhang, Xin Liu, Xuehua Jiang and Ling Wang

1. Tables

Table S1. Summary of HPLC-MS/MS conditions used in sample analysis.

Analyte	Analytical column	Mobile phase	Gradient pattern % B/time (min)	Mass transition m/z Q1–Q3	Collision energy (V)
Paclitaxel	CAPCELL PAK C18, 50 × 2.00 mm 5um	A:0.2% formic acid in water; B: acetonitrile	20/0-1, 20-95/1-2, 95/2-3, 20/3-4	854.2-285.9	23.2
6α-Hydroxy paclitaxel				870.8-286.1	26.7

Table 2. Oligonucleotides used in this study.

Oligo name	Source	Forward (5' to 3')	Reverse (5' to 3')	Genebank accession number
CYP2C8	human	GGCCACTTTCTAGATAAGAATG	TGATAGCAGATCGGCAGCCA	NM_000770.3
CYP3A4	human	GCAGGAGGAAATTGATGCAGTT	GTCAAGATACTCCATCTGTAGCACAGT	NM_017460.6
GAPDH	human	GTCAACGGATTTGGTCGTATTG	TGTAGTTGAGGTCAATGAAGGG	NM_002046.7
Cyp2c6	rats	TCAGCAGGAAAACGGATGTG	AATCGTGGTCAGGAATAAAAATAACTC	NM_001013904.2
Cyp2c11	rats	CCCTGAGGACTTTTGGGATGGGC	GGGGCACCTTTGCTCTTCCTC	NM_019184.2
Cyp2c22	rats	GGCCACTTTCTAGATAAGAATG	TGATAGCAGATCGGCAGCCA	NM_138512.2
Cyp3a1	rats	CCATCACGGACACAGAAATG	CTTTCCCCATAATCCCCACT	NM_013105.2
Cyp3a2	rats	AGTGGGGATTATGGGGAAAG	CAATGATGGGGAACATCTCC	NM_153312.3
Gapdh	rats	GGCACAGTCAAGGCTGAGAATG	ATGGTGGTGAAGACGCCAGTA	NM_017008.4

2. Figures

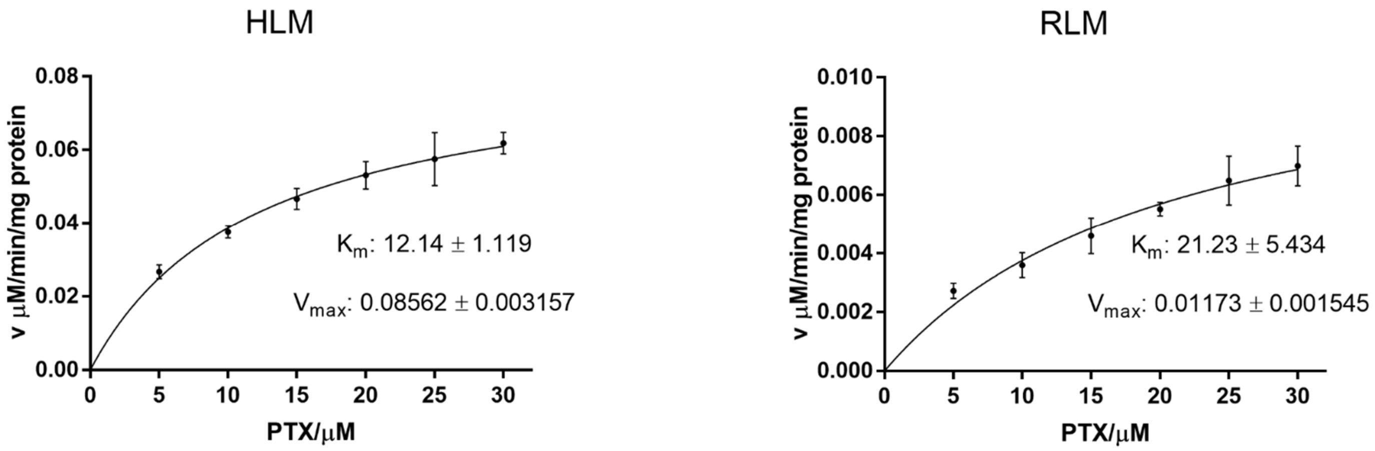

Figure S1. Kinetic plots for 6α′-hydroxytaxol formation velocity versus substrate concentration in HLM and RLM. Each data point represents the mean of triplicate. The lines represent the best fit to the data using nonlinear regression.

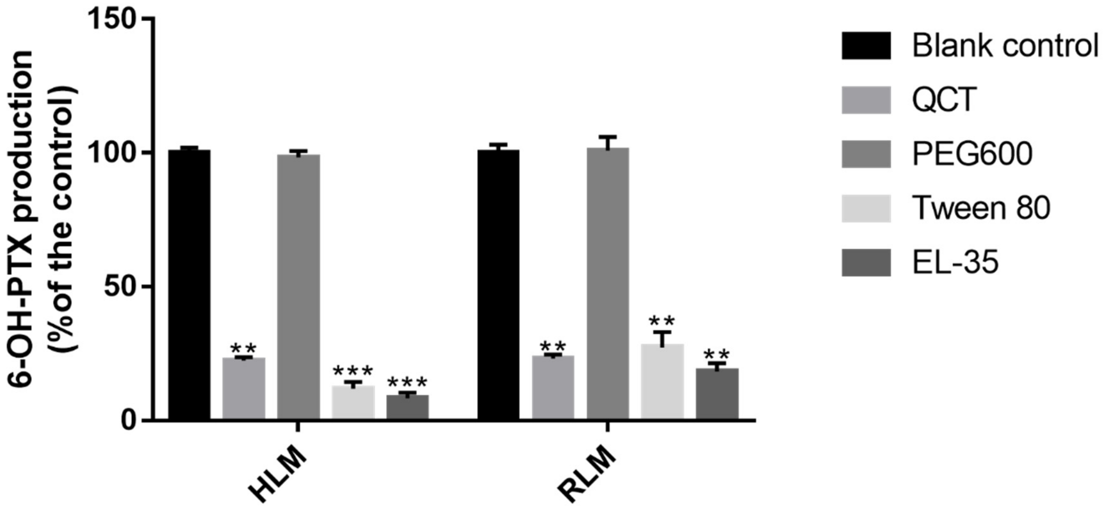

Figure S2. Effects of various PEs on 6α′-hydroxylation of PTX in HLM and RLM. PTX concentrations were 10 μM and 20μM for HLM and RLM, respectively. QCT (10uM) was served as the positive control. Each data point represents the mean of triplicate. **p < 0.01; ***p < 0.001 compared with blank control.

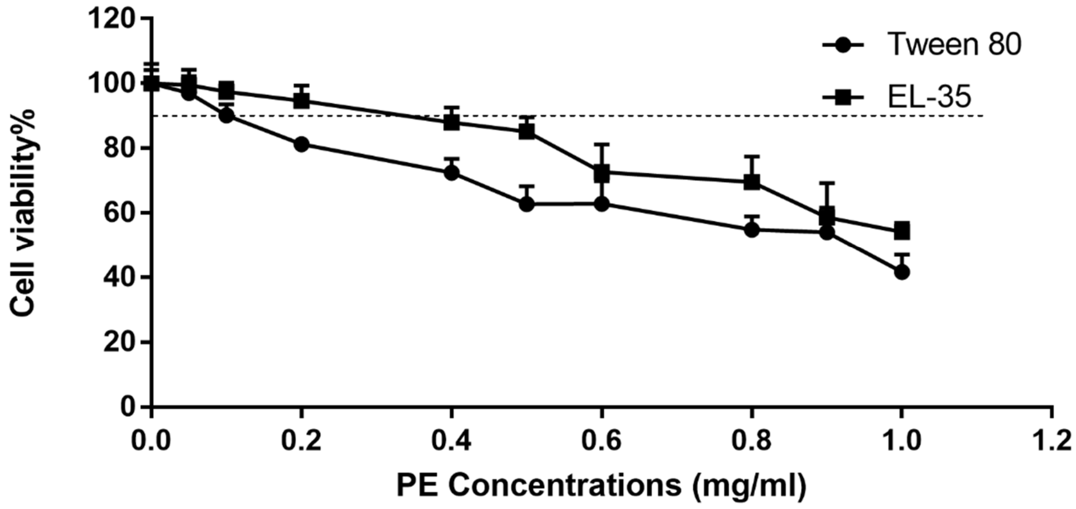

Figure S3. Cell viability rates calculated by MTT assay. HepG2 cells were treated with the indicated concentrations of Tween 80 and EL-35 for 24 h, respectively. Data were expressed as the mean ± S.D. (n=3 replicates/treatment).

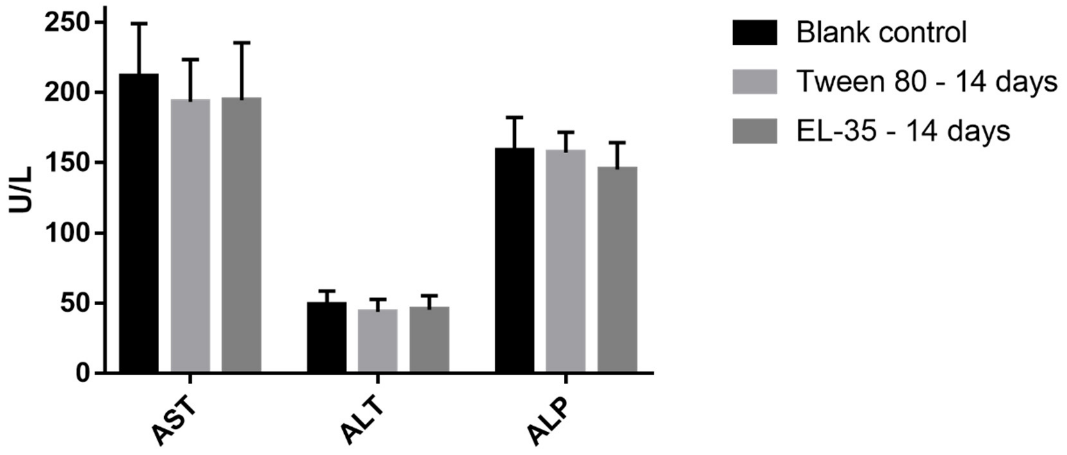

Figure S4. Effects of Tween 80 and EL-35 on the rats' liver function after multiple doses. The main serum indexes (AST/ALT/ALP) of liver function in Wistar rats were determined after multiple administration of PEs (Tween 80, EL-35) for 14 days. Data were expressed as the mean ± S.D. (n=6 replicates/treatment).

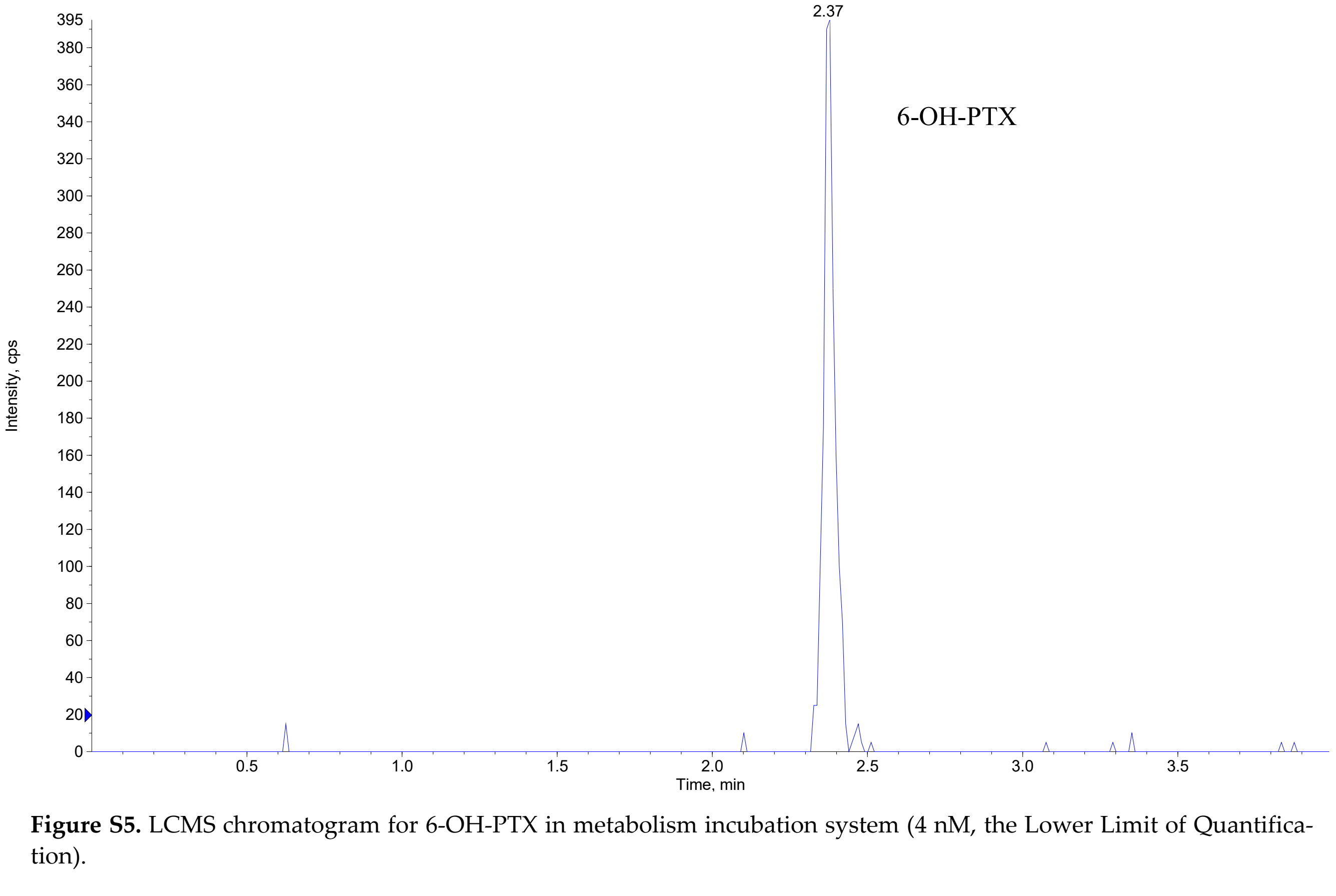

Figure S5. LCMS chromatogram for 6-OH-PTX in metabolism incubation system (4 nM, the Lower Limit of Quantification).

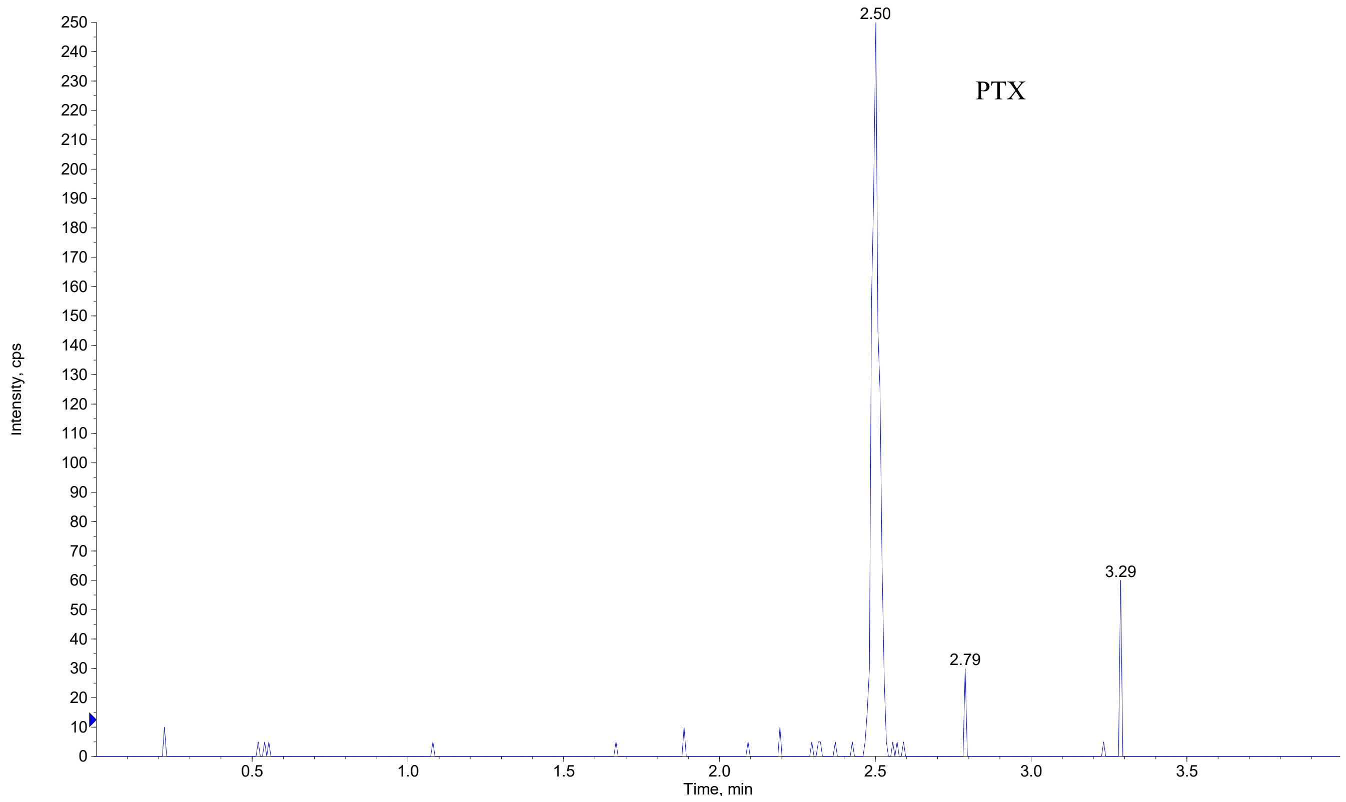

Figure S6. LCMS chromatogram for PTX in rat plasma (10 ng/mL, the Lower Limit of Quantification).

3. Estimation of EL-35 exposure in human:

Information from the label of Taxol:

1. Each mL of solution contains 6 mg PTX and 527 mg EL-35. (That is, 527 mg EL-35/6 mg PTX)
2. Taxol administration:
a. administrated intravenously over 3 hours at a dose of 175 mg/m^2
b. administrated intravenously over 24 hours at a dose of 135 mg/m^2

Here, we are assuming a female ovarian cancer patient who weighs 57.3 kg and is 155.8 cm tall (according to the average body weight and height of Chinese women reported by National Health Commission of China (2020)), so that her body surface area (BSA) should be 1.56 m^2. The dosing regimen she may receive is 273.0 mg PTX (i.v. > 3 hours) or 210.6 mg PTX (i.v. > 24 hours) according to Equation 1. So that, the patient's exposure level of EL-35 in the body under the two regimens is **418.5 mg/kg** (i.v. > 3 hours) or **322.8 mg/kg** (i.v. > 24 hours) according to Equation 2.

$$BSA \times D1 \qquad (Equation\ 1)$$

D1 in Equation 1 is the dose of 175 mg/m^2 or 135 mg/m^2.

$$\frac{D2 \times 527mg}{6mg \times W_{human}} \qquad (Equation\ 2)$$

D2 in Equation 2 represents the dosage calculated by Equation 1 (273.0 mg or 210.6 mg); W_{human} represents the average body weight of Chinese women (57.3 kg).

The EL-35 dose set in this study (430 mg/kg) equivalent to **71.5 mg/kg** in human according to Equation 3.

$$D3 \times \left(\frac{\overline{W_{rat}}}{W_{human}}\right)^{0.33} \qquad (Equation\ 3)$$

In Equation 3, D3 represents the EL-35 dose set in this study (430 mg/kg); $\overline{W_{rat}}$ represents the average weight of rats in this study (about 250 g); W_{human} represents the average body weight of Chinese women (57.3 kg).

(The equivalent dose conversion is based on FDA Guidance for Industry ***Estimating the Maximum Safe Starting Dose in Initial Clinical Trials for Therapeutics in Adult Healthy Volunteers***. https://www.fda.gov/regulatory-information/search-fda-guidance-documents/estimating-maximum-safe-starting-dose-initial-clinical-trials-therapeutics-adult-healthy-volunteers)
The estimated exposure of EL-35 in human is about 4.5~5.9 times of the dose we set in this study.
